# Sea Urchin-like NiCo_2_O_4_ Catalyst Activated Peroxymonosulfate for Degradation of Phenol: Performance and Mechanism

**DOI:** 10.3390/molecules29010152

**Published:** 2023-12-26

**Authors:** Chunguang Chen, Junkai Zhang, Jia Liu, Jiani Li, Shuo Ma, Aishui Yu

**Affiliations:** 1 Department of Chemistry, School of Materials and Chemistry, University of Shanghai for Science and Technology, Shanghai 200093, China; 223353287@st.usst.edu.cn (J.Z.); 223353218@st.usst.esu.cn (J.L.); 2135051101@st.usst.edu.cn (J.L.); 2235070108@st.usst.edu.cn (S.M.); 2 Shanghai Key Laboratory of Molecular Catalysis and Innovative Materials, Collaborative Innovation Center of Chemistry for Energy Materials, Department of Chemistry, Institute of New Energy, Fudan University, Shanghai 200438, China

**Keywords:** PMS activation, non-radical activation, radical activation, sea urchin-like NiCo_2_O_4_, synergistic effect

## Abstract

How to efficiently activate peroxymonosulfate (PMS) in a complex water matrix to degrade organic pollutants still needs greater efforts, and cobalt-based bimetallic nanomaterials are desirable catalysts. In this paper, sea urchin-like NiCo_2_O_4_ nanomaterials were successfully prepared and comprehensively characterized for their structural, morphological and chemical properties via techniques, such as X-ray diffraction (XRD), transmission electron microscopy (TEM), scanning electron microscopy (SEM), X-ray photoelectron spectroscopy (XPS), among others. The sea urchin-like NiCo_2_O_4_ nanomaterials exhibited remarkable catalytic performance in activating PMS to degrade phenol. Within the NiCo_2_O_4_/PMS system, the removal rate of phenol (50 mg L^−1^, 250 mL) reached 100% after 45 min, with a reaction rate constant k of 0.091 min^−1^, which was 1.4-times higher than that of the monometallic compound Co_3_O_4_/PMS system. The outstanding catalytic activity of sea urchin-like NiCo_2_O_4_ primarily arises from the synergistic effect between Ni and Co ions. Additionally, a comprehensive analysis of key parameters influencing the catalytic activity of the sea urchin-like NiCo_2_O_4_/PMS system, including reaction temperature, initial pH of solution, initial concentration, catalyst and PMS dosages and coexisting anions (HCO_3_^−^, Cl^−^, NO_3_^−^ and humic acid), was conducted. Cycling experiments show that the material has good chemical stability. Electron paramagnetic resonance (EPR) and quenching experiments verified that both radical activation (SO_4_^•−^, ^•^OH, O_2_^•−^) and nonradical activation (^1^O_2_) are present in the NiCo_2_O_4_/PMS system. Finally, the possible degradation pathways in the NiCo_2_O_4_/PMS system were proposed based on gas chromatography–mass spectrometry (GC-MS). Favorably, sea urchin-like NiCo_2_O_4_-activated PMS is a promising technology for environmental treatment and the remediation of phenol-induced water pollution problems.

## 1. Introduction

Recently, the increasing water pollution caused by the discharge of large quantities of industrial wastewater has aroused concern and worry. In particular, industrial wastewater containing phenol and Rhodamine B is considered persistent, difficult to degrade and composed of hazardous organic pollutants [1,2]. This can adversely affect the quality of human life by way of the food chain and environmental cycles. Therefore, developing economical and efficient technologies is urgent to remove these organic pollutants from water bodies. Among the many wastewater treatment technologies, advanced oxidation processes (AOPs) based on peroxymonosulfate (PMS) are considered as promising technologies for the degradation of organic pollutants because of their simplicity, high efficiency, good reproducibility and reduced secondary pollution [3].

Previous studies have explored various strategies for the degradation of organic pollutants in water by PMS-based AOPs [4,5,6], where most of the degradation mechanisms have been attributed to independent radical or non-radical pathways. The radical pathway refers to the fact that PMS can generate reactive free radicals with high redox potentials, including SO_4_^•−^ and ^•^OH, through certain external conditions (acoustic, optical, electrical, thermal, transition metals and their oxides, etc.), which can lead to the complete mineralization of organic pollutants [7,8,9]. The non-radical pathway relies on reactive oxygen species other than free radicals and oxidative processes, such as ^1^O_2,_ and direct electron transfer from organic electron donors to the PMS on the catalyst surface [10]. The radical pathway and non-radical pathway each have advantages and disadvantages. Radical-based AOPs with high oxidation potentials have excellent degradation performance, but side reactions and the corresponding by-products usually occur due to the non-targeted attack of free radicals [11,12]. In contrast, the non-radical pathway is more selective for certain organics, such as electron-donating compounds [13]. However, effective degradation by non-radical processes only occurs for electron-donating contaminants (e.g., aniline) and not for electron-absorbing contaminants (e.g., benzoic acid) [13]. It has been shown that the simultaneous action of radical and non-radical pathways on pollutants is more effective than single pathway treatment due to the synergistic effect. Therefore, the design of a catalyst that can efficiently activate PMS with the presence of both radical and non-radical pathways is very promising in the field of PMS-based AOPs.

The literature suggests that cobalt is the most powerful element for PMS activation in AOPs [14,15]. Several stable cobalt oxides (e.g., CoO and Co_3_O_4_) are frequently used as activators for PMS [16,17]. Nevertheless, the leaching of toxic divalent cobalt ions, the small specific surface area and few active sites limit the practical application of monometallic cobalt oxides [18]. The introduction of other polyvalent transition metal elements may alter the morphology and structure of the material in comparison to monometallic cobalt oxides, which not only helps to improve the catalytic performance of the materials but also reduces the leaching of cobalt ions [19,20]. Therefore, it is a wise strategy to improve the above defects and maintain the excellent catalytic activity by introducing other transition metal elements.

In recent years, a broad range of cobalt-based bimetallic catalysts have been widely used in AOP because of their excellent catalytic activity. Yu et al. [21] successfully synthesized MgCo_2_O_4_ spinel via a hydrothermal method and tested its catalytic performance for PMS activation using bisphenol A (BPA) as the target pollutant. The results showed that the MgCo_2_O_4_/PMS system could effectively degrade 99.6% of BPA within 10 min at pH 7.2. In this case, the tetrahedral Mg^2+^ may make MgCo_2_O_4_ more stable and promote the redox cycle of Co^2+^/Co^3+^, which ultimately leads to the degradation of BPA through both radical and non-radical pathways. A.Q.K. Nguyen et al. [22] reported that CoWO_4_ nanoparticles synthesized by adjusting the PH during hydrothermal synthesis can efficiently degrade 4-chlorophenol by activated PMS. The experimental results showed that the excellent performance of the CoWO_4_ catalyst (CoWO_4_-10) synthesized at pH 10 is attributed to its large specific surface area, the good charge transfer properties and the synergistic effect between Co and W ions. Ultimately, the organic compounds were rapidly degraded relying on both free radical and non-free radical pathways. In addition, NiCo_2_O_4_ is also an excellent semiconductor material for various catalytic applications, and its higher conductivity helps in electron transfer [23,24,25].

Based on the above factors, sea urchin-like NiCo_2_O_4_ was rapidly synthesized via a simple hydrothermal method and thermal treatment for the catalytic degradation of phenol in water by activated PMS. The effects of important factors, such as catalyst and PMS dosage, initial solution concentration, initial pH, reaction temperature and coexisting anions and humic acid (HA), on the catalytic activity of sea urchin-like NiCo_2_O_4_ were explored. The synergistic effect of Co^2+^-Co^3+^/Ni^3+^-Ni^2+^ in the sea urchin-like NiCo_2_O_4_ promoted the generation of more reactive oxygen species (ROS). The quenching experiments verified that both radical and non-radical pathways participated in the activation of PMS. Additionally, the GC-MS explored the intermediate products of the degradation of phenol in the NiCo_2_O_4_/PMS system. The present study suggests that sea urchin-like NiCo_2_O_4_-activated PMS is a promising technology for environmental treatment and remediation in response to phenol-induced water pollution problems.

## 2. Results and Discussion

### 2.1. Characterizations of the Sea Urchin-like NiCo_2_O_4_ Catalysts

**Figure 1:**
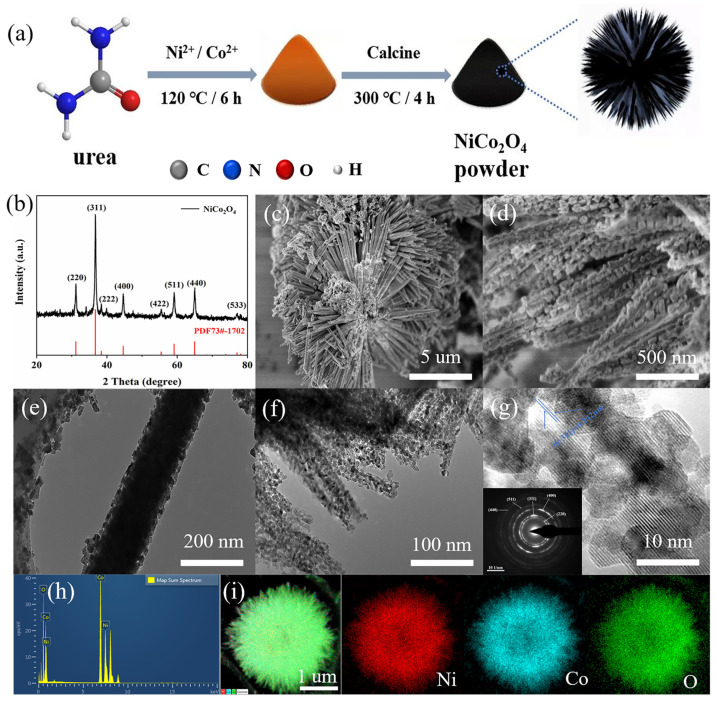
(**a**) Schematic diagram of the preparation for the sea urchin-like NiCo_2_O_4_ catalysts. (**b**) XRD pattern of the sea urchin-like NiCo_2_O_4_. (**c**,**d**) The SEM images of the sea urchin-like NiCo_2_O_4_. (**e**,**f**) The TEM images of the sea urchin-like NiCo_2_O_4_. (**g**) TEM image of the sea urchin-like NiCo_2_O_4_ (inset: selected area electron diffraction pattern). (**h**) EDS element content image. (**i**) EDS mapping images of sea urchin-like NiCo_2_O_4_.

The sea urchin-like NiCo_2_O_4_ was rapidly synthesized using a simple hydrothermal and thermal treatment method (Figure 1a). The crystal phase compositions of the synthesized NiCo_2_O_4_, Co_3_O_4_ and NiO were characterized by XRD (Figure 1b and Figure S1a,b). The synthesized NiCo_2_O_4_ exhibits characteristic diffraction peaks, where the diffraction peaks at 31.2°, 36.7°, 38.4°, 44.6°, 55.4°, 59.1°, 65.0° and 77.0° correspond to the (220), (311), (222), (400), (422), (511), (440) and (533) crystalline planes of NiCo_2_O_4_ (JCPDS 73-1702), respectively. Similarly, the XRD patterns of the synthesized Co_3_O_4_ and NiO corresponded to their standard spectra. These results indicated that NiCo_2_O_4_ bimetallic oxides as well as monometallic oxides of Co and Ni were successfully prepared [26].

The morphology and structure of the sea urchin-like NiCo_2_O_4_ were described using SEM and TEM. According to Figure 1c,d, the synthesized NiCo_2_O_4_ appears as sea urchin-like microspheres with uniform size (~8 μm). The microspheres are composed of an orderly combination of needle-like structures with a solid interior, and the needle-like structures constituting the sea urchin-like NiCo_2_O_4_ microspheres are formed by nanoparticles. Accordingly, TEM images of NiCo_2_O_4_ (Figure 1e,f) further confirmed that the microspheres were composed of an orderly combination of a needle-like structure with a diameter of about 200 nm, which was accumulated by nanoparticles. The BET results (Figure S1c,d) further verified that NiCo_2_O_4_ is a mesoporous material based on the obvious H3 hysteresis loop, and its specific surface area is 40.15 m^2^ g^−1^. Obviously, the unique structure and large surface area can provide a certain number of reaction sites for the surface reactions, and the sea urchin-like structure can maintain the structural stability of the material [26,27,28]. As a comparison, the morphologies of monometallic oxide NiO and Co_3_O_4_ are microsphere structures with relatively uniform size and rod-shaped, respectively (Figure S2). Furthermore, according to the TEM image of NiCo_2_O_4_ (Figure 1g), the lattice stripes with a spacing of 0.242 nm correspond to the (311) crystalline surface of NiCo_2_O_4_. Selected-area electron diffraction in the inset of Figure 1g also shows well-defined diffraction rings, which coincide with the aforementioned XRD results of the NiCo_2_O_4_ material. EDS analyses determined the presence of nickel and cobalt metals (Figure 1h). Additionally, the EDS mapping image of the sea urchin-like NiCo_2_O_4_ material showed a uniform distribution of Ni, Co and O (Figure 1i), indicating the successful synthesis of bimetallic oxides.

**Figure 2:**
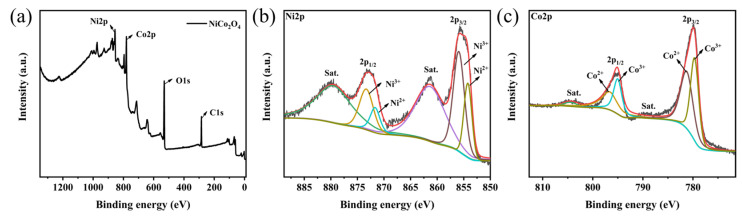
XPS diagram of the sea urchin-like NiCo_2_O_4_ material (**a**) full spectrum, (**b**) Ni 2p, (**c**) Co 2p.

The chemical composition and surface electronic valence states of the sea urchin-like NiCo_2_O_4_ catalyst were further studied by XPS experiments, as shown in Figure 2. The XPS full spectrum in Figure 2a shows that the sample contains Ni, Co and O and no miscellaneous peaks of other elements, which is in perfect agreement with the XRD test results. The XPS fine spectrum after Ni fitting (Figure 2b) shows two satellite peaks and four binding energy fitting peaks at 854.2 eV, 855.9 eV, 872.2 eV and 874.1 eV. Among them, two main peaks at 854.2 eV and 872.2 eV proved the presence of Ni^2+^, while two main peaks at 855.9 eV and 874.1 eV proved the presence of Ni^3+^. Similarly, the XPS fine spectrum of Co (Figure 2c), likewise, shows two satellite peaks and four binding energy-fitted peaks at 779.3 eV, 780.9 eV, 794.9 eV and 795.5 eV. The two main peaks at 780.9 eV and 795.5 eV prove the presence of Co^2+^, while the two main peaks at 779.3 eV and 794.9 eV prove the presence of Co^3+^. Based on the above experimental results, it can be speculated that on the surface of the sea urchin-like NiCo^2^O^4^ catalyst, the interaction of Ni and Co with different valence states can generate additional electron holes, which is conducive to the transfer of electrons, thus improving the catalytic activity of the sea urchin-like NiCo_2_O_4_ material [28].

### 2.2. Catalytic Performance

**Figure 3:**
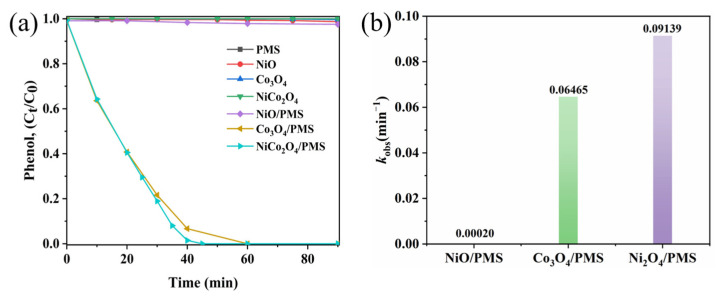
(**a**) Phenol removal in different systems. (**b**) The degradation rate constants (k) of phenol in different systems.

To evaluate the catalytic activity of the sea urchin-like NiCo_2_O_4_ catalyst, phenol was selected as the contaminants of this experiment. The degradation of phenol by PMS autoxidation and the adsorption of phenol by different kinds of catalysts were investigated through controlled experiments. As shown in Figure 3a, under the condition of PMS alone, the removal of phenol was less than 1% in 90 min, which indicated that PMS had no obvious degradation effect on phenol, and the degradation effect of PMS autoxidation was negligible. The absence of PMS, NiO, Co_3_O_4_ and sea urchin-like NiCo_2_O_4_ catalysts all showed similar adsorption effects on phenol, which were less than 1%, and the above results indicated that the adsorption effect of the catalysts was also negligible. In addition, three catalytic degradation systems, NiO/PMS, Co_3_O_4_/PMS and sea urchin-like NiCo_2_O_4_/PMS, were explored for phenol degradation under the same experimental conditions. The complete degradation of phenol in the sea urchin-like NiCo_2_O_4_/PMS system was 45 min, the complete degradation of phenol in the Co_3_O_4_/PMS system was 60 min and the removal of phenol in the NiO/PMS system was only about 2.5% in 90 min. The above experimental results show that the introduction of Ni substantially enhances the catalytic activity of pure cobalt oxides. This is mainly due to the synergistic effect between Ni and Co, which accelerates the electron transfer rate and, thus, improves the catalytic activity of the material [29]. Additionally, the mineralization of phenol and RhB reached 67.5% and 62.7%, as displayed in Figure S3a,b, respectively, indicating that significant quantities of organic compounds were degraded to inorganic carbides in the NiCo_2_O_4_/PMS system. In summary, the sea urchin-like NiCo_2_O_4_ exhibits the best catalytic activity.

Furthermore, the kinetics for the degradation of phenol using different catalyst/PMS systems also confirmed the remarkable catalytic performance of the NiCo_2_O_4_/PMS system (Figure 3b). The degradation rate constants of the sea urchin-like NiCo_2_O_4_/PMS system (k = 0.09139 min^−1^) were 1.4-times and 450-times higher than those of the Co_3_O_4_/PMS system (k = 0.06465 min^−1^) and NiO/PMS system (k = 0.00020 min^−1^), respectively. The result suggests that the doping of Ni plays an important role in activating PMS to degrade phenol. Moreover, Table S1 lists the catalytic properties of some catalysts in the literatures compared with the NiCo_2_O_4_ catalyst in this work for phenol degradation [30,31,32,33]. As can be seen from the table, the sea urchin-like NiCo_2_O_4_/PMS system shows excellent catalytic performance in phenol degradation. Overall, the great catalytic activity of the sea urchin-like NiCo_2_O_4_ was attributed to the synergistic effect between nickel and cobalt, which accelerated the electron transfer rate and accelerated the phenol degradation reaction [34].

### 2.3. Influence of Reaction Parameters on Phenol Removal

#### 2.3.1. Effect of Catalyst and PMS Dosages

**Figure 4:**
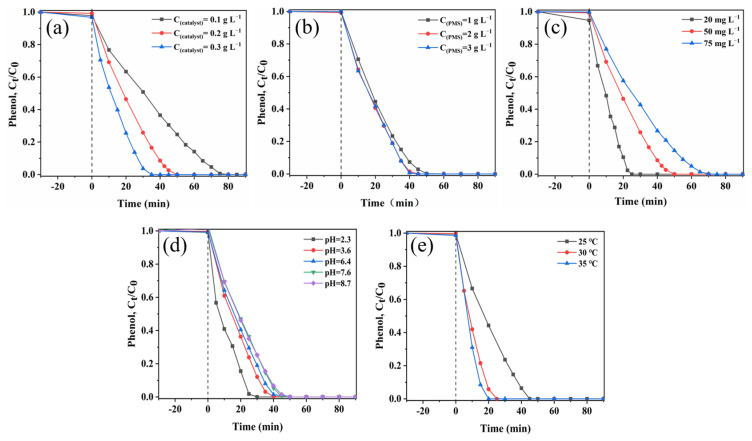
Influence of (**a**) catalyst dosages. (**b**) PMS dosages. (**c**) Phenol concentrations. (**d**) pH of the solution. (**e**) Temperatures on phenol degradation in the sea urchin-like NiCo_2_O_4_/PMS system.

Firstly, the effects of the dosages of sea urchin-like NiCo_2_O_4_ in the catalyst/PMS for the phenol removal rate were investigated. According to Figure 4a and Figure S4a, the higher the amount of sea urchin-like NiCo_2_O_4_ present in the system, the higher the k value of the phenol degradation reaction, which accelerated the effective degradation of the pollutant. As the catalyst dosage was increased from 0.1 g L^−1^ to 0.2 g L^−1^, the phenol removal rate increased from 75% to 100% in 50 min. As the catalyst content in the system continued to increase to 0.3 g L^−1^, phenol was completely decomposed in 35 min. Based on Figure S4a, the k value increased from the initial 0.05139 min^−1^ to 0.0745 min^−1^ and 0.09859 min^−1^ when the dosage of sea urchin-like NiCo_2_O_4_ was increased from 0.1 g L^−1^ to 0.2 g L^−1^ and 0.3 g L^−1^. The higher value of k may be attributed to the fact that more catalysts provided more reactive active sites, which accelerated the PMS activation and, hence, promoted the decomposition process of phenol [35]. In summary, a 0.2 g L^−1^ catalyst concentration was selected for subsequent study based on the practical application and economic efficiency.

Figure 4b and Figure S4b show the influence of the dosage of PMS on phenol degradation. In the sea urchin-like NiCo_2_O_4_/PMS system, the removal rate of phenol increased sequentially from 92.7% to 98.5% and 99.4% within 40 min as the PMS concentration was increased sequentially from 1 g L^−1^ to 2 g L^−1^ and 3 g L^−1^. The degradation kinetic constants of phenol revealed that the k value increased sequentially from the initial 0.07384 min^−1^ to 0.09139 min^−1^ and 0.10416 min^−1^ (Figure S4b). Normally, an increase in PMS concentration increases the amounts of active substances in the sea urchin-like NiCo_2_O_4_/PMS system, which, in turn, improves the efficiency of pollutant degradation. However, the degradation efficiency of phenol and the kinetic constant k of the reaction changed very little when the PMS concentration was increased from 2 g L^−1^ to 3 g L^−1^. This was caused by the fact that the non-activated PMS reacts with active species (SO_4_^•−^ and ^•^OH) by self-bursting, thus becoming a limiting factor for the degradation reaction [36]. Therefore, an optimum PMS concentration of 2 g L^−1^ was determined as the actual dosage in this study.

#### 2.3.2. Effect of Initial Phenol Concentration

Figure 4c and Figure S4c show the effect of different initial concentrations of phenol (between 20 mg L^−1^–75 mg L^−1^) on the degradation process. Both the degradation efficiency of phenol and the rate constant of the reaction decreased with an increasing phenol concentration. At a low phenol concentration (20 mg L^−1^), 100% degradation of phenol could be achieved within 25 min. As the concentration of phenol further increased to 50 mg L^−1^ and 75 mg L^−1^, the time required for its complete degradation increased to 50 min and 70 min, respectively. The corresponding k values decreased from 0.14122 min^−1^ to 0.07450 min^−1^ and 0.05621 min^−1^, respectively. The main reason is that the number of active species is certain and, thus, a longer degradation time is needed with a higher concentration of phenol solution. In addition, more intermediates adsorbed on the catalyst surface are generated in highly concentrated phenol solutions, thus preventing PMS from binding to the catalyst active sites.

#### 2.3.3. Effect of Initial pH

The activation efficiency of PMS in advanced oxidation techniques (AOPs) is depended on the initial pH of the solution. According to Figure 4d and Figure S4d, the effect of different pH values on phenol degradation was ascertained. As the pH increased from 2.3 to 3.6, 6.4, 7.6 and 8.7, the phenol removal decreased sequentially, and the k value of the degradation reaction decreased sequentially from an initial value of 0.13892 min^−1^ to 0.12461 min^−1^, 0.09139 min^−1^, 0.06666 min^−1^ and 0.06238 min^−1^, respectively. The result indicated that the degradation reaction was biased towards acidic conditions. In addition, according to the results of previous studies, the interaction between the catalyst surface and phenol molecules can be improved by adjusting the solution pH, which can accelerate the decomposition of pollutants through the formation of a variety of active substances on the catalyst surface [37,38]. The surface of sea urchin-like NiCo_2_O_4_ is positively charged within acidic solutions, which effectively attracts HSO_5_^−^ near the material to generate more SO_4_^•−^ [37]. However, HSO_5_^−^ cannot be stabilized in alkaline environments and reacts as shown in Equation (1), resulting in a greatly reduced phenol removal rates [39].

(1) $$2HSO_{5}{}^{-}+2OH^{-}\to 2SO_{4}{}^{2-}+O_{2}+H_{2}O$$

#### 2.3.4. Effect of Temperature

The effect of reaction temperatures (25 °C, 30 °C and 35 °C) on the phenol removal was investigated in the sea urchin-like NiCo_2_O_4_/PMS system. As shown in Figure 4e, the time required for the complete degradation of phenol was reduced to 45 min, 25 min and 20 min at 25 °C, 30 °C and 35 °C, respectively. According to Figure S4e, the rate constant k of the reaction increases significantly with increasing reaction temperature, and the k value at 35 °C (k = 0.15630 min^−1^) is more than twice of the k value at 25 °C (k = 0.06364 min^−1^). The experimental results showed that phenol and PMS molecules were more active at higher temperatures and, thus, provide more opportunities for the PMS to collide with the active sites of the catalyst [40]. In addition, the activation energy (Ea) of the reaction is obtained using the Arrhenius equation (Equation (21)). The Ea in the sea urchin-like NiCo_2_O_4_/PMS system was calculated to be 68.89 kJ mol^−1^ by fitting the equation (ln k = −8.286/T + 25.135, R^2^ = 0.871). The Ea value in the system is greater than that of the diffusion-controlled reaction (10^−13^ kJ mol^−1^), suggesting that the degradation of phenol in the system is attributable to the surface-mediated inner chemical reaction instead of the mass transmission [41]. Therefore, higher temperatures would increase the activation of PMS by sea urchin-like NiCo_2_O_4_ to produce more reactive oxygen species, thus improving the catalytic degradation efficiency.

As mentioned earlier, both temperature and the catalyst have a large effect on the Ea during the catalytic degradation reaction of phenol. The activation energies for the degradation of phenol by several catalytic systems are listed in Table S2 [42,43]. The data shown in Table S2 indicate that the activation energy of the sea urchin-like NiCo_2_O_4_/PMS system for phenol degradation is lower than that of most of the catalyst/PMS systems in the literature, which suggests that the sea urchin-like NiCo_2_O_4_/PMS has the advantage of good catalytic degradation of phenol.

#### 2.3.5. Influences of Inorganic Ions and Humic Acid

Large amounts of inorganic anions and humic acids (HAs) are universally present in natural aquatic systems. Undoubtedly, they may affect the degradation process of pollutants due to the reaction with free radicals. To investigate the practical value of the NiCo_2_O_4_/PMS system in environmental aquatic systems, the effects of inorganic anions (HCO_3_^−^, Cl^−^, NO_3_^−^) and HA on the degradation performance of phenol were systematically investigated in this experiment. The specific experimental results and analysis are as follows.

**Figure 5:**
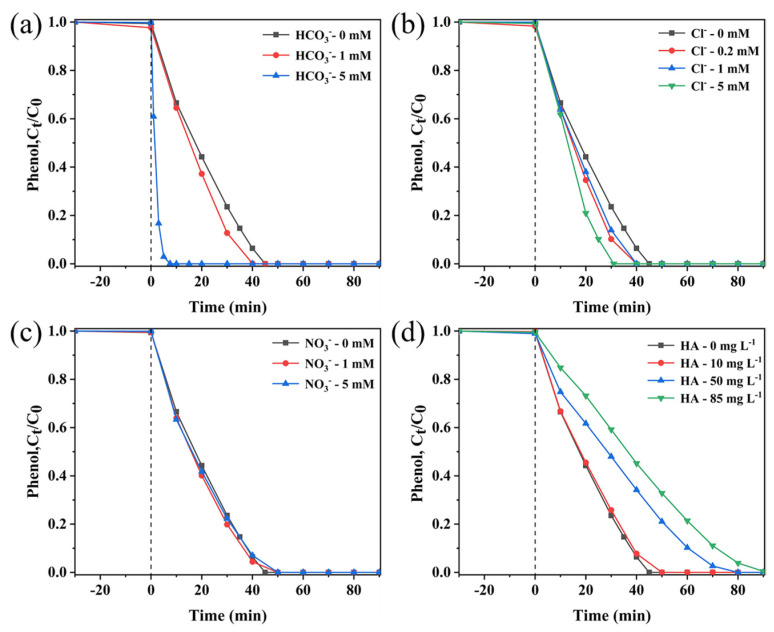
Influences of (**a**) HCO_3_^−^, (**b**) Cl^−^, (**c**) NO_3_^−^ and (**d**) HA on phenol removal efficiency in sea urchin-like NiCo_2_O_4_/PMS system. Conditions: (phenol) = 50 mg L^−1^, (catalyst) = 0.2 g L^−1^, (PMS) = 2 g L^−1^, T = 25 °C.

Effect of coexisting HCO_3_^−^: according to Figure 5a, the complete degradation time of phenol was shortened from the initial 45 min to 40 min and 8 min with increasing the concentration of HCO_3_^−^ in the system from 0 mM to 1 mM and 5 mM, respectively. Therefore, HCO_3_^−^ can promote the activation of PMS to cause more ROS, thereby promoting the degradation of phenol [44,45].

Effect of coexisting Cl^−^: the phenol removal rate was significantly enhanced with the increase in the Cl^−^ concentration from 0 mM to 5 mM, and the time for complete degradation was shortened from 45 to 30 min (Figure 5b). According to the literature, Cl^−^ can activate PMS to generate HOCl (Equation (2)), which can selectively react with electron-rich phenols to accelerate phenol degradation. The degradation of phenol is also facilitated by the reaction between Cl^−^ and ^•^ClOH^−^ to form ^•^Cl_2_^−^ (Equations (3) and (4)) [46,47].

(2) $$Cl^{-}+HSO_{5}{}^{-}\to SO_{4}{}^{2-}+HOCl$$

(3) Cl−+OH•→ClO•H−

(4) Cl−+ClO•H−→Cl2−•+OH−

Effect of coexisting NO_3_^−^: As shown in Figure 5c, NO_3_^−^ only shows a slight inhibitory effect on the phenol degradation process. The complete degradation time of phenol was prolonged from 45 min to 50 min when the concentration of NO_3_^−^ was increased from 0 mM to 5 mM, which indicated that the presence of NO_3_^−^ had a slight inhibitory effect on the degradation process of phenol. The reason could be that NO_3_^−^ reacts with reactive species (SO_4_^•−^ and ^•^OH) (Equations (5) and (6)) [37] to produce less reactive NO_3_^•^ (2.0–2.2 V vs. NHE).

(5) $$NO_{3}{}^{-}+SO_{4}{}^{•-}\to NO_{3}{}^{•}+SO_{4}{}^{2-}$$

(6) NO3−+OH•→NO3•+OH−

Effect of coexisting HA: HA, as an important component of natural organic substances, cannot be ignored in the actual pollutant management process. Therefore, the effect of HA on phenol degradation was also investigated in this study (Figure 5d). With increasing the HA concentration (0 mg L^−1^ to 85 mg L^−1^) in the sea urchin-like NiCo_2_O_4_/PMS degradation system, the removal efficiency of phenol was gradually inhibited. It can be seen that the presence of HA has a serious inhibitory effect on the degradation of phenol, and this inhibitory effect becomes more and more obvious with the increase in HA concentration, which is attributed to the following reasons: (1) HA usually acts as a radical scavenger competing for the active radicals. (2) HA is more readily adsorbed onto the surface of the catalyst through its own hydroxyl and carboxyl groups and blocks the reaction sites [44,48].

On the whole, both HCO_3_^−^ and Cl^−^ can promote phenol degradation, the presence of NO_3_^−^ has almost no effect on the phenol degradation, and HA can inhibit phenol degradation in the sea urchin-like NiCo_2_O_4_/PMS system. Research has shown that non-radical reactions are lower in sensitivity to co-existing anions in water compared to free radical reactions, indicating that the nonradical pathway participated in the reaction in the sea urchin-like NiCo_2_O_4_/PMS system [49].

### 2.4. Reusability and Stability of the Sea Urchin-like NiCo_2_O_4_

**Figure 6:**
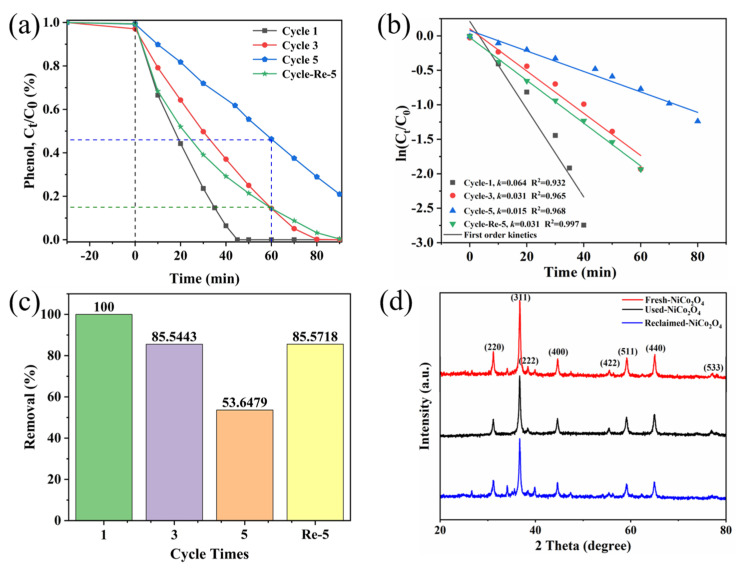
(**a**) C_t_/C_0_-t diagram of phenol degradation in the cycling experiment. (**b**) The first-order kinetic simulation of the reaction in the cycling experiment. (**c**) Histogram of the phenol removal rate at 60 min in the cycling experiment. (**d**) Fresh and used XRD patterns of sea urchin-like NiCo_2_O_4_.

The stability and reusability of catalysts are crucial in practical applications. Therefore, the reusability of the sea urchin-like NiCo_2_O_4_ catalyst and the crystal phase composition before and after use were further investigated (Figure 6). According to Figure 6a,c, the catalytic activity of sea urchin-like NiCo_2_O_4_ showed an overall decreasing trend after five cycling experiments, which may be attributed to the adsorption of some intermediates on the catalyst surface, which could not be removed by filtration and washing with water. Subsequently, after the fourth cycle, the material was recalcined at 300 °C for 4 h, and the degradation efficiency of the catalyst was found to increase from 53.6% to 85.6%, indicating that calcination helps to remove the adhering substances on the catalyst surface and fully expose its active sites. Furthermore, Figure 6b shows the variation in the k value for each cycle reaction, which is consistent with the trend of the phenol removal rate in each cycle. The above results reveal that the sea urchin-like NiCo_2_O_4_ material can be efficiently recycled several times after recalcination. In addition, comparing the XRD patterns of the used and fresh catalysts (Figure 6d), no obvious change in the XRD curves was found, which further confirmed the good chemical stability and durability of the sea urchin-like NiCo_2_O_4_.

### 2.5. Catalytic Mechanism and Phenol Degradation Pathway

**Figure 7:**
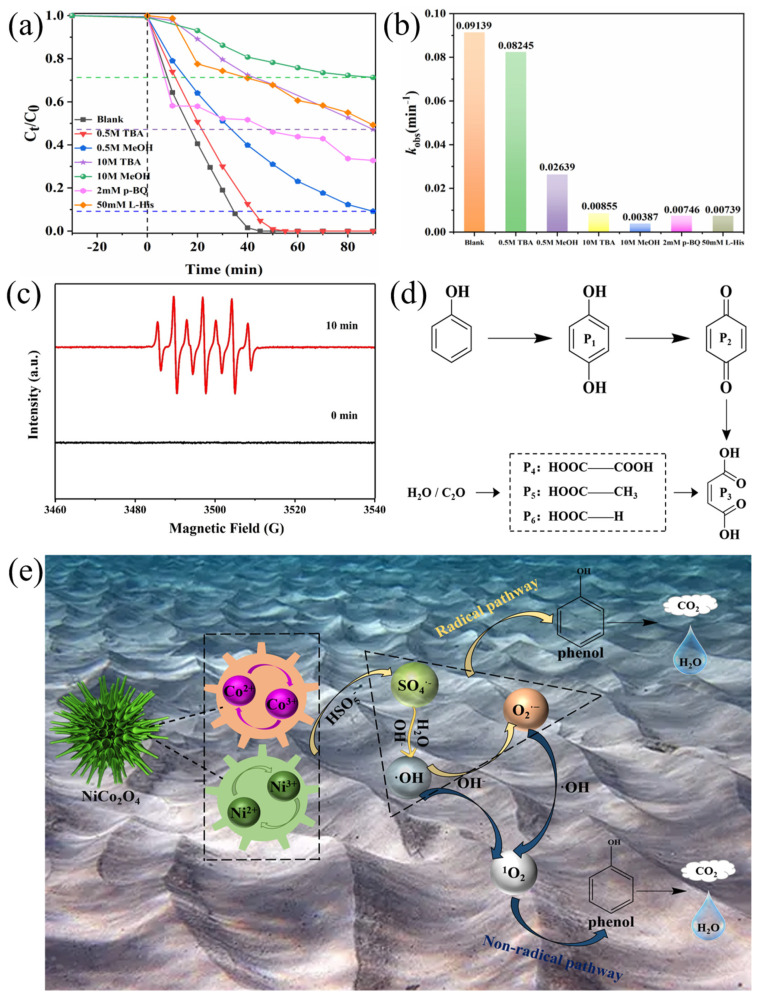
(**a**) The effect of various radical quenchers (TBA, MeOH, p-BQ and L-His) on phenol degradation in NiCo_2_O_4_/PMS system. (**b**) Histogram of rate constants for different quenching reactions. (**c**) EPR spectra of DMPO-SO_4_^•−^ and DMPO-^•^OH at the first and the 10th minute. (**d**) Proposed decomposition pathway of phenol. (**e**) PMS activation mechanism of the sea urchin-like NiCo_2_O_4_/PMS system (conditions: (phenol) = 50 mg L^−1^, (catalyst) = 0.2 g L^−1^, (PMS) = 2 g L^−1^, T = 25 °C).

To investigate the catalytic mechanism, a series of quenching experiments was performed to determine the types and contributions of reactive oxygen species in the sea urchin-like NiCo_2_O_4_/PMS system. As reported in the literature [50], it is known that MeOH is usually used to scavenge SO_4_^•−^ and ^•^OH, and TBA is a bursting agent for ^•^OH. From Figure 7a, phenol was completely removed after 45 min without adding any quenching agent, whereas the degradation of phenol was only about 90% and 29% with the addition of 0.5 M and 10 M MeOH, respectively. Correspondingly, the k value was reduced from 0.09139 min^−1^ (not added) to 0.02639 min^−1^ (added 0.5 M methanol) and 0.00387 min^−1^ (added 10 M methanol) (Figure 7b). When 0.5 M TBA was added to the sea urchin-like NiCo_2_O_4_/PMS system, the removal of phenol remained almost constant, although the k value of the degradation reaction decreased slightly. With increasing the concentration of TBA to 10 M, the phenol removal rate reduced to about 53%, and the k value decreased to 0.00855 min^−1^ (Figure 7a,b). The results showed that the addition of either a TBA or MeOH quencher significantly inhibited phenol degradation, and the inhibition of phenol degradation gradually increased with the increase in the concentration of the TBA or MeOH quencher, indicating that both SO_4_^•−^ and ^•^OH were involved in the activation of PMS. In addition, EPR experiments (Figure 7c) further verified that both SO_4_^•−^ and ^•^OH were generated in the NiCo_2_O_4_/PMS system based on a typical seven-line EPR signal [46]. Notably, phenol degradation is still not completely inhibited within 10 M MeOH, suggesting that other ROS are also involved in the degradation reaction. For this reason, p-benzoquinone (p-BQ) and lev histidine (L-His) were used as O_2_^•−^ and _1_O^2^ bursting agents, respectively, to determine whether they were involved in the phenol degradation process [51,52]. As shown in Figure 7a,b, both the phenol removal rate and degradation rate constant k decreased dramatically after the addition of 2 mM p-BQ and 50 mM L-His, which suggests that O_2_^•−^ and _1_O^2^ also play an essential role in phenol degradation.

Based on the above XPS analysis of the catalyst (Figure 2) and quenching experiments (Figure 7a), the possible reaction mechanism of phenol degradation by sea urchin-like NiCo_2_O_4_-activated PMS can be proposed as follows. First, the ≡Co and ≡Ni ions combine with H_2_O as Lewis acid/base sites to form ≡Co-^−^OH and ≡Ni-^−^OH. Subsequently, the ≡Co^2+^-^−^OH and ≡Ni^2+^-^−^OH species on the surface of the urchin-like NiCo_2_O_4_ activate PMS to form surface-bound SO_4_^•−^ (Equations (7) and (9)). Meanwhile, the formed ≡Co^3+^-^−^OH and ≡Ni^3+^-^−^OH react with PMS to regenerate more ≡Co^2+^-^−^OH and ≡Ni^2+^-^−^OH species (Equations (8) and (10)), respectively. Furthermore, according to the XPS analysis, redox reactions occur between Co^3+^/Co^2+^ and Ni^3+^/Ni^2+^ on the surface of the sea urchin-like NiCo_2_O_4_ material (Equation (11)), which may enhance the electron transfer effect and accelerate PMS activation. The action of these two redox pairs is similar to the Fenton reaction based on the Harber–Weiss cycle [53]. Namely, the synergistic effect between the Ni and Co can facilitate the activation of PMS to generate SO_4_^•−^. Based on the analysis of previous studies [54,55], the partial SO_4_^•−^ directly participates in the decomposition of phenol, and the remaining SO_4_^•−^ can react with H_2_O/OH^−^ to form ^•^OH (Equations (12) and (13)). Subsequently, a portion of ^•^OH directly participates in the decomposition process, and the remaining ^•^OH is involved in the generation of O_2_^•−^ (Equations (14)–(17)). Finally, the nonradical species of ^1^O_2_ comes from the O_2_^•−^ (Equation (18)). Since the above reactions proceed in a cyclic manner, phenol in solution is under constant assault by the radical pathway based on the ROSs (SO_4_^•−^, ^•^OH, O_2_^•−^) and nonradical pathway (^1^O_2_) until they are completely decomposed to CO_2_ and H_2_O (Equation (19)). The reaction mechanism of phenol degradation in the sea urchin-like NiCo_2_O_4_/PMS system is shown schematically in Figure 7e.

(7) ≡Co2+−OH−+HSO5−→≡Co3+−OH−+SO4•−+OH−

(8) ≡Co3+−OH−+HSO5−→≡Co2+−OH−+SO5•−+H+

(9) ≡Ni2+−OH−+HSO5−→≡Ni3+−OH−+SO4•−+OH−

(10) ≡Ni3+−OH−+HSO5−→≡Ni3+−OH−+SO5•−+H+

(11) $$Ni^{2+}+Co^{3+}\to Ni^{3+}+Co^{2+}$$

(12) SO4•−+H2O→OH•+SO42−+H+

(13) SO4•−+OH−→OH•+SO42−

(14) $${}^{•}OH+OH^{-}\to \;O^{•-}+H_{2}O$$

(15) $${}^{•}OH+O^{•-}\to HO_{2}{}^{-}$$

(16) $${}^{•}OH+HO_{2}{}^{-}\to OH^{-}+HO^{2•}$$

(17) $$HO^{2•}\to \;O_{2}{}^{•-}+H^{+}$$

(18) O2•−+OH• →O21+OH−

(19) $$SO_{4}{}^{•-}{/}^{•}OH/\;O_{2}{}^{•-}{/}^{1}O_{2}+phenol\;\to \;intermediates\;\to \;CO_{2}+H_{2}O$$

To further investigate the degradation pathway of phenol in the sea urchin-like NiCo_2_O_4_/PMS system, GC-MS was employed to measure the intermediates produced during phenol degradation. A few compounds, like dihydroxy benzene and benzoquinone, were initially determined (Figure S6). Furthermore, the UV spectrograms at different time points during the phenol degradation process verified that some intermediate products were produced (Figure S5). Based on the present experimental results and previous studies [56], possible degradation pathways for phenol were proposed (Figure 7d). First, the para or neighboring sites of the hydroxyl (^−^OH) on the ring are assaulted by ROS to produce dihydroxy benzene, which then continues to be oxidized by ROS to produce p-benzoquinone, whose benzene ring and carbon–carbon double bond are destroyed by ROS and converted into oxalic acid, which is finally broken down into CO_2_ and H_2_O by a decarboxylation process. The high TOC removal rate (67.5%) in Figure S3a indirectly indicated that the final products were transformed into CO_2_ and H_2_O, which finally achieved the effective mineralization of the pollutants.

## 3. Materials and Methods

### 3.1. Chemicals and Materials

CoCl_2_·6H_2_O, NiCl_2_·6H_2_O, urea, peroxymonosulfate (PMS), phenol, Rhodamine B (RhB), HCl, NaOH, NaHCO_3_, NaCl, NaNO_3_, humic acid (HA), tert-butanol (TBA), methanol (MeOH) and acetonitrile (liquid chromatography pure) were provided by Shanghai Titan Scientific Co., Ltd. (Shanghai, China). All the chemicals were analytical-grade reagents and above and used without further purification. Deionized water was used in all experiments.

### 3.2. Preparation of the Sea Urchin-like NiCo_2_O_4_ Catalysts

The sea urchin-like NiCo_2_O_4_ catalysts were prepared and synthesized by hydrothermal and thermal treatment method. To start with, 4 mmol of CoCl_2_·6H_2_O, 2 mmol of NiCl_2_·6H_2_O and 6 mmol of urea were dissolved in deionized water and stirred well. The mixed solution was then transferred to hydrothermal reactor and heated at 120 °C for 6 h, and purple-pink powder was obtained. Finally, the sea urchin-like NiCo_2_O_4_ catalysts were obtained by calcining the prepared purple-pink powder at 300 °C for 4 h under an air atmosphere. For the purpose of comparison, nickel and cobalt monometallic oxides were synthesized separately using the above method but without the addition of nickel and cobalt sources.

### 3.3. Degradation Experiments

The catalytic degradation experiments were performed in a quartz reactor containing 250 mL phenol solution and placed on a magnetron stirrer equipped with a thermal sensor and a waterbath kettle (the speed of the magneton was set to 500 rpm). Firstly, a quantitative amount of catalyst was added to the phenol solution for half an hour to reach the adsorption and desorption equilibrium of the material. A quantitative amount of the oxidant PMS was then added to the contaminant solution to begin removal of the phenol. At the set time interval, 1 mL of sample solution was taken out, filtered by 0.22 μm filter membrane and quenched by the addition of 0.5 mL methanol, followed by UV and HPLC tests, respectively. Experimental parameters of different inquiry experiments can be adjusted according to the principle of a single variable. Diluted HCl and NaOH solutions were used to regulate the pH value of the original solution. After the degradation reaction, the catalyst in the solution was filtered and reused. With the degradation rate of phenol as the index, the stability and reusability of the catalyst were tested. In addition, to compare the catalytic performance of different catalysts, the same degradation experiments were carried out based on the synthesized single metal oxide catalysts. In quenching experiment, different quenchers were added to phenol solution. The hydroxyl radical (^•^OH) was quenched by TBA; ^•^OH and sulfate radical (SO_4_^•−^) were quenched by MeOH. Super oxygen radicals (O_2_^•−^) and ^1^O_2_ were quenched by para-benzoquinone (p-BQ) and L-Histidine (L-His), respectively. The main reactive oxygen species (ROS) in the system were determined by comparing the removal rates of phenol.

### 3.4. Characterizations

X-ray diffraction (XRD, D max/RB diffractometer, Rigaku Corporation, Tokyo, Japan) with Cu Kα radiation (λ = 1.5406 Å) was carried out to measure the crystal structure and purity of the materials. The microscopic morphologies and structural features of the synthesized materials were obtained via scanning electron microscopy (SEM, Shimadzu S4800, Hitachi Corporation, Hitachi, Japan) with an X-ray energy-dispersive spectrometer (EDS) and transmission electron microscope (TEM, JEM-200CX, JEOL, Akishima, Japan). The Brunauer–Emmett–Teller (BET) specific surface area, pore size distribution and pore volume of the catalysts were characterized via using a physisorption instrument (JW-ZK222). The elemental composition and chemical valence states of the catalysts were recorded by X-ray photoelectron spectroscopy (XPS, ESCALAB 250Xi, Thermo Scientific, Waltham, MA, USA). During the phenol’s degradation process, total organic carbon analyzer (TOC, Shimadzu-VCSH, Kyoto, Japan) was used to determine the TOC content in the solution. The concentration of phenol was determined by HPLC (Elite-EClassical 3100, Dalian, China) on a C-18 HPLC column (5 μm, 4.6 × 250 mm) with an ultraviolet detection wavelength of 270 nm. Typically, acetonitrile and UP water were exploited as mobile phase (40% organic phase and 60% aqueous phase). The flow rate was set to 1 mL min^−1^. The effects of the catalysts on phenol degradation efficiency were monitored by ultraviolet-visible absorption spectroscopy (UV–Vis, TU-1810PC, Beijing, China). In order to better identify the active oxygen species (ROSs, SO_4_^•−^, ^•^OH, O_2_^•−^ and ^1^O_2_) during catalytic reaction, the effects of ROS were determined by the free radical quenching experiments and electron paramagnetic resonance trials (EPR, brooke-a300). The intermediates produced at different time points in the degradation process were identified by gas chromatography–mass spectrometry (GC-MS, Shimadzu-QP2020, Kyoto, Japan).

### 3.5. Calculation Methods

The degradation kinetics of phenol was fitted by the quasi-first-order reaction equation, and its apparent reaction rate constant k was calculated according to Equation (20):

(20) $$ln\;\left(C_{t}/C_{0}\right)=-kt$$

where t is the reaction time, and C_t_ and C_0_ represent phenol concentration at time t and initial phenol concentration, respectively [57]. With t as the abscissa and ln (C_t_/C_0_) as the ordinate, the slope obtained after fitting is the apparent reaction constant k of the system (unit: min^−1^).

Furthermore, the reaction activation energy (Ea) during the phenol degradation reaction could be calculated through the Arrhenius equation (Equation (21)).

(21) ln k=−Ea/RT+ln A

where k represents the reaction rate constant (min^−1^), Ea is activation energy (kJ mol^−1^), R is the molar gas constant (8.314 J mol^−1^ K^−1^), T represents thermodynamic temperature (K) and A is a constant [58].

## 4. Conclusions

In this study, the sea urchin-like NiCo_2_O_4_ microspheres were successfully synthesized using a simple hydrothermal method followed by thermal treatment, applied for the phenol degradation in the activation of PMS. Thanks to the synergistic redox cycle between Ni and Co ion and its stable structure of the sea urchin-like NiCo_2_O_4_ microspheres, the catalyst material showed good catalytic performances in activating PMS for the degradation of phenol. In the sea urchin-like NiCo_2_O_4_/PMS system, phenol solution could be completely removed within 45 min with a good mineralization rate, which is attributed to the activation of radical species. The sea urchin-like NiCo_2_O_4_ exhibits enhanced PMS activation across a broad spectrum of PH values. In modeling environmental aquatic systems, both HCO_3_^−^ and Cl^−^ can promote phenol degradation, and the presence of NO_3_^−^ has almost no effect on the phenol degradation in the sea urchin-like NiCo_2_O_4_/PMS system, which is due to the participation of non-radical species. In addition, the sea urchin-like NiCo_2_O_4_ microspheres exhibited extraordinary reusability. The quenching experiments and EPR experiments confirmed that both radical species (SO_4_^•−^, ^•^OH and O_2_^•−^) and non-radical species (^1^O_2_) are important reactive oxygen species in the sea urchin-like NiCo_2_O_4_/PMS system. Furthermore, the degradation pathway of phenol was propounded based on the detected intermediates via GC–MS. This study suggests that sea urchin-like NiCo_2_O_4_-activated PMS is a promising technology for environmental treatment and remediation for phenol-induced water pollution problems.

## Data Availability

The data presented in this study are available in the Supplementary Materials.

## Supplementary Materials

The following supporting information can be downloaded at: https://www.mdpi.com/article/10.3390/molecules29010152/s1, Figure S1: (a) XRD pattern of (a) Co_3_O_4_. (b) NiO. (c) N_2_ adsorption/desorption isotherms of the sea urchin-like NiCo_2_O_4_. (d) pore size distribution image of the sea urchin-like NiCo_2_O_4_; Figure S2: (a) The SEM images of (a,b) Co_3_O_4_, (c,d) NiO; Figure S3: (a) TOC removal rate of phenol in NiCo_2_O_4_/PMS degradation system. (b) TOC removal rate of RhB in NiCo_2_O_4_/PMS degradation system; Figure S4: First kinetic simulation diagram of phenol degradation under the influence of reaction parameters. (a) catalyst dosages, (b) PMS dosages, (c) initial phenol concentrations, (d) initial pH, (e) reaction temperatures; Figure S5: The SEM image of used sea urchin-like NiCo_2_O_4_ catalysts; Figure S6: UV-vis spectral changes of phenol in the sea urchin-like NiCo_2_O_4_/PMS degradation system; Figure S7: GC (a–c) chromatogram for the phenol degradation in the sea urchin-like NiCo_2_O_4_/PMS system. (d–f) MS spectrum of the intermediates from phenol degradation; Table S1: Comparison with other catalysts for phenol degradation; Table S2: Comparison of Ea for phenol degradation by different catalyst/PMS systems. References [30,31,32,33,42,43] are cited in the supplementary materials.
