# Supplementary material for: Sea Urchin-like NiCo2O4 Catalyst Activated Peroxymonosulfate for Degradation of Phenol: Performance and Mechanism
Source: Molecules. 2023 Dec 26;29(1):152. doi: 10.3390/molecules29010152 (PMC10780213; doi:10.3390/molecules29010152)
Supplement: Supplementary file 1 [file molecules-29-00152-s001.zip › molecules-2719497-supplementary.pdf]

Supplementary Materials

Sea Urchin-like NiCo2O4 Catalyst Activated Peroxymonosulfate for Degradation of Phenol: Performance and Mechanism

Chunguang Chen [1,*], Junkai Zhang [1], Jia Liu [1], Jiani Li [1], Shuo Ma [1] and Aishui Yu [2,*]

1 Department of Chemistry, School of Materials and Chemistry, University of Shanghai for Science and Technology, Shanghai 200093, China; 223353287@st.usst.edu.cn (J.Z.); 223353218@st.usst.esu.cn (J.L.); 2135051101@st.usst.edu.cn (J.L.); 2235070108@st.usst.edu.cn (S.M.)

2 Shanghai Key Laboratory of Molecular Catalysis and Innovative Materials, Collaborative Innovation Center of Chemistry for Energy Materials, Department of Chemistry, Institute of New Energy, Fudan University, Shanghai 200438, China

* Correspondence: cgchen19@usst.edu.cn (C.C.); asyu@fudan.edu.cn (A.Y.)

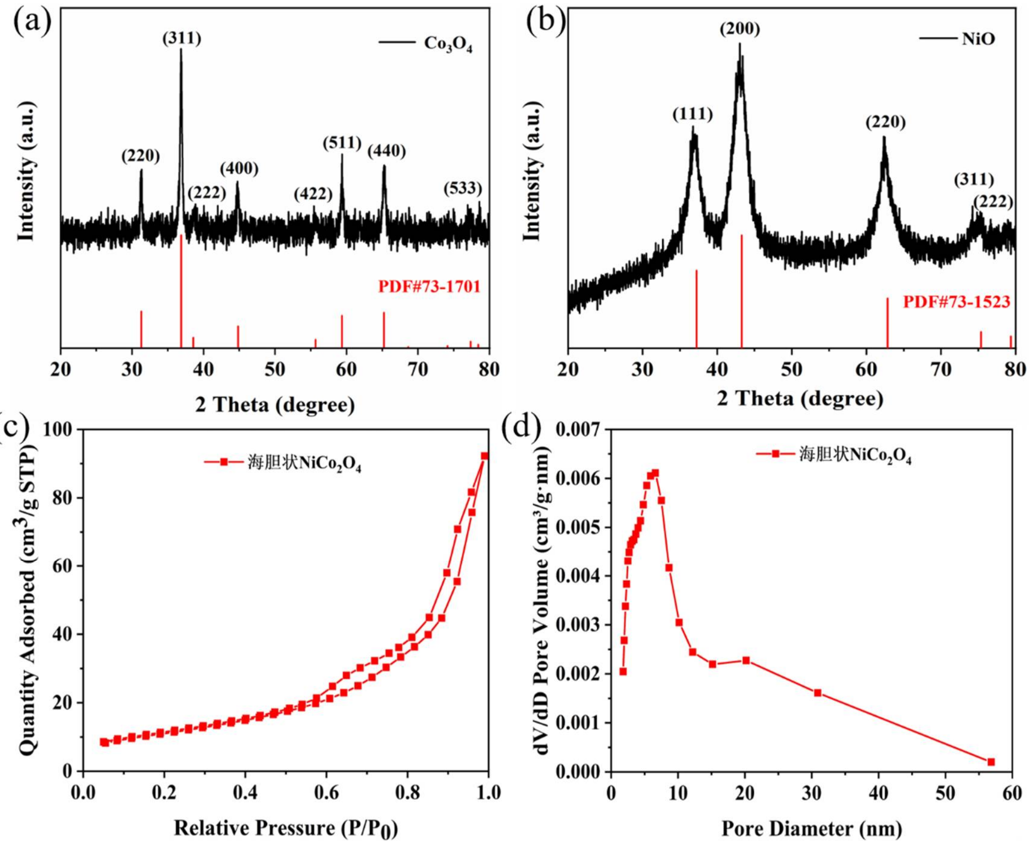

Figure S1. (a) XRD pattern of (a) Co_3O_4. (b) NiO. (c) N_2 adsorption/desorption isotherms of the sea urchin-like $NiCo_2O_4$. (d) pore size distribution image of the sea urchin-like $NiCo_2O_4$.

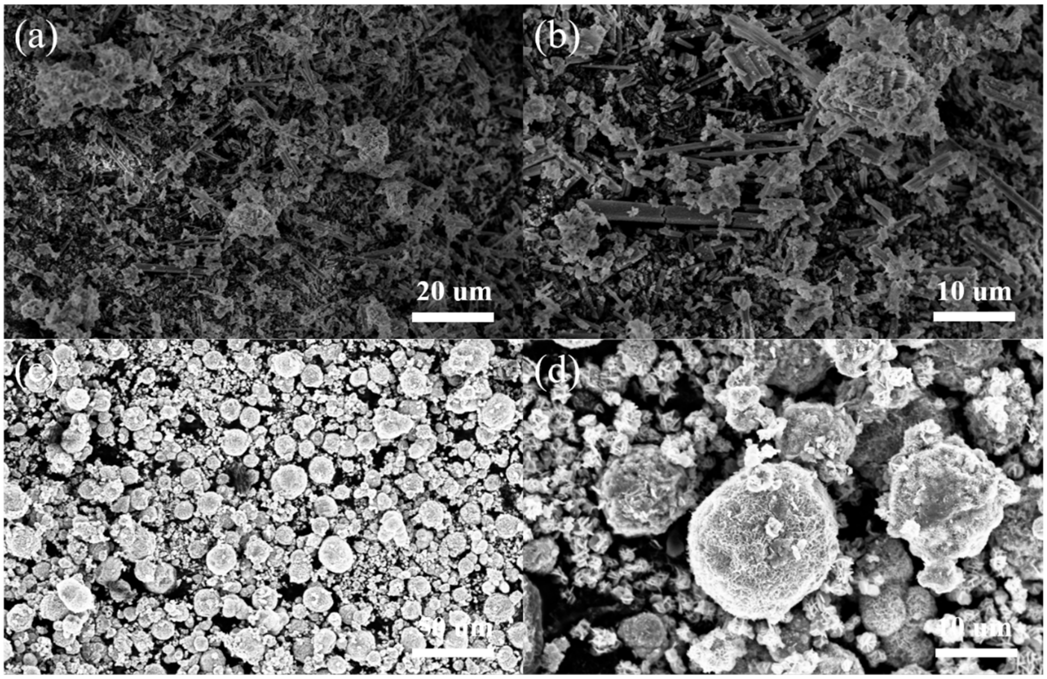

Figure S2. (a) The SEM images of (a, b) Co_3O_4, (c, d) NiO.

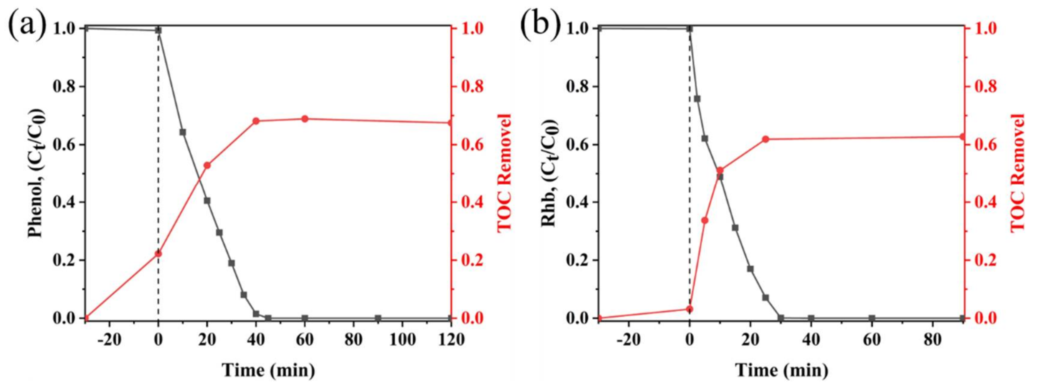

Figure S3. (a) TOC removal rate of phenol in $NiCo_2O_4$/PMS degradation system. (b) TOC removal rate of RhB in $NiCo_2O_4$/PMS degradation system.

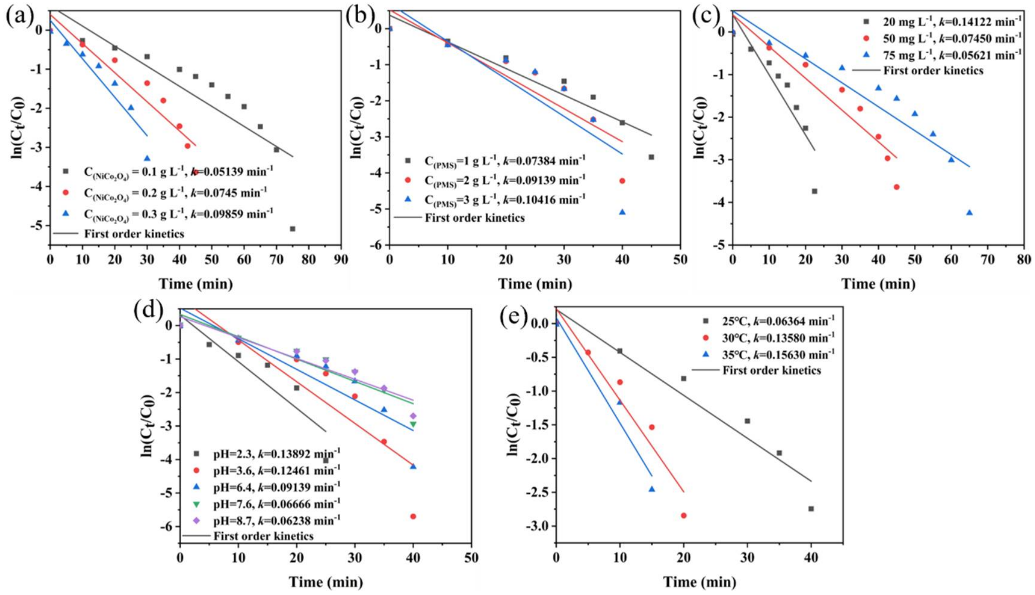

Figure S4. First kinetic simulation diagram of phenol degradation under the influence of reaction parameters. (a) catalyst dosages, (b) PMS dosages, (c) initial phenol concentrations, (d) initial pH, (e) reaction temperatures.

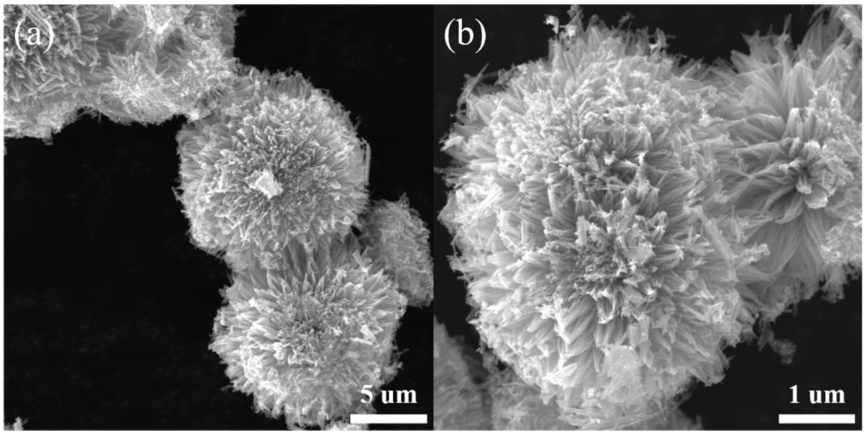

Figure S5. The SEM image of used sea urchin-like $NiCo_2O_4$ catalysts.

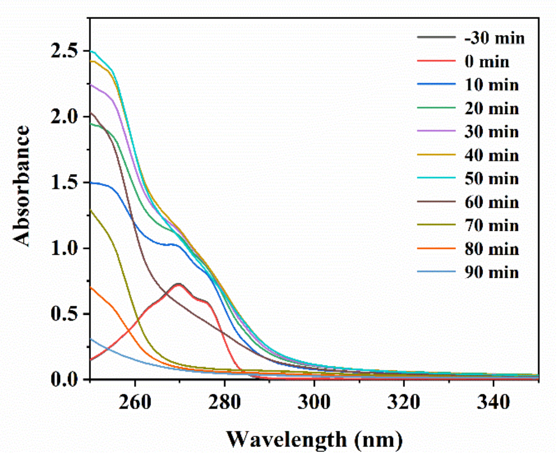

Figure S6. UV-vis spectral changes of phenol in the sea urchin-like $NiCo_2O_4$/PMS degradation system.

According to the UV spectrograms of phenol degradation at different time points, it can be seen that the characteristic absorption peak of phenol at 270 nm in the visible region shows a tendency to be strong first and then gradually disappear. The deformation of the characteristic peak of phenol is due to the formation of some intermediate products with strong absorbance during the reaction. With the degradation reaction, phenol was decomposed by various reactive oxygen species in the sea urchin-like $NiCo_2O_4$/PMS system, and finally CO_2 and H_2O were generated, and the characteristic peaks disappeared finally.

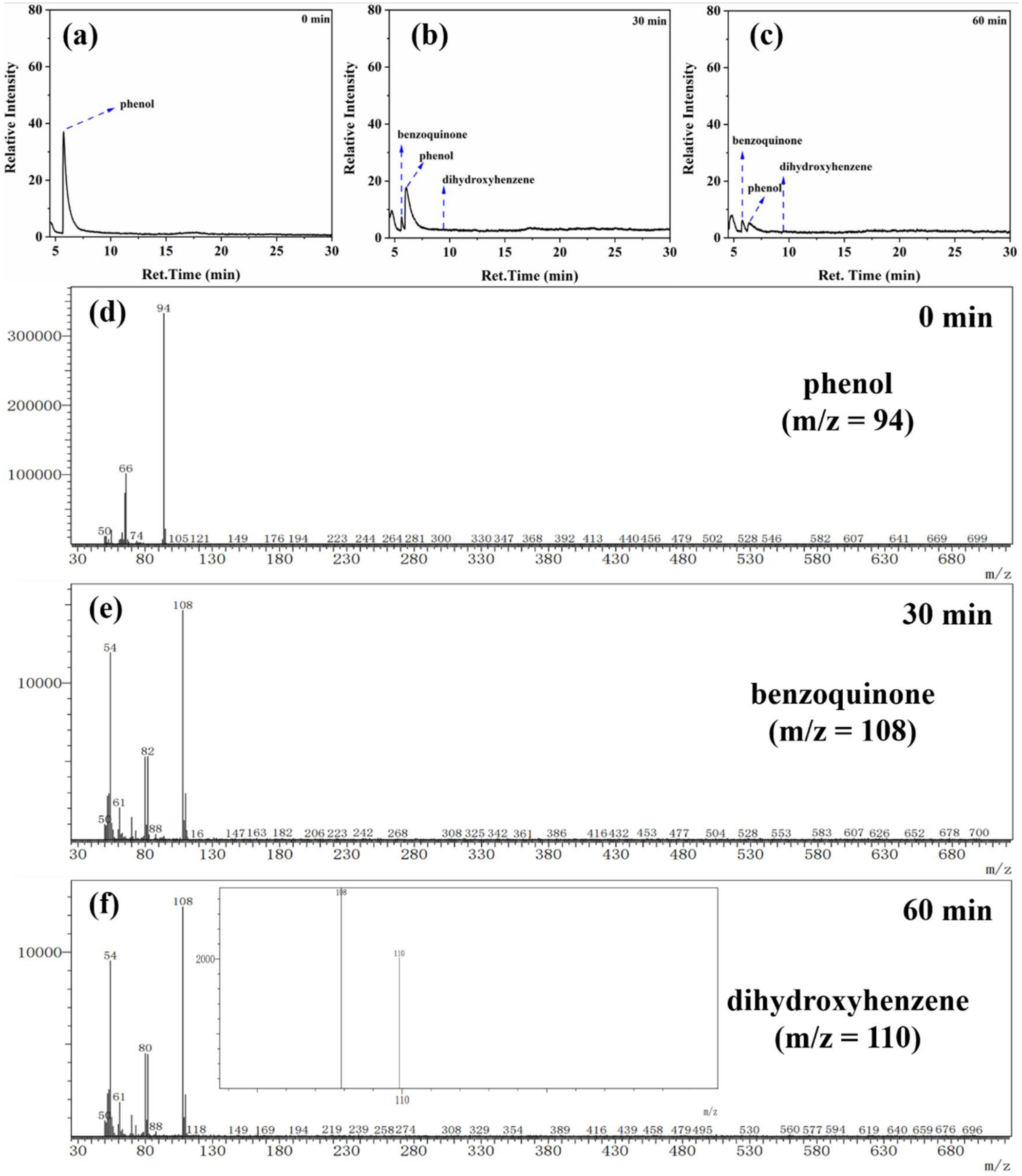

Figure S7. GC (a-c) chromatogram for the phenol degradation in the sea urchin-like $NiCo_2O_4$/ PMS system. (d-f) MS spectrum of the intermediates from phenol degradation.

Table S1. Comparison with other catalysts for phenol degradation.

Type of catalyst	Concentration of catalyst	Concentration of PMS	Concentration of phenol	Efficiency (%)	k (min^{-1})	Reference
CoMgAl-LDH	0.3 g L^{-1}	3 mM	0.1 mM (20mL)	100 (60min)	------	[1]
RuO_2/Al_2O_3	0.4 g L^{-1}	2.0 g L^{-1}	50 mg L^{-1} (500 mL)	100 (60min)	------	[2]
$CeVO_4$	1.0 g L^{-1}	2.0 g L^{-1}	100 mg L^{-1} (50 mL)	100 (80min)	0.043	[3]
$CuFe_2O_4/MnO_2$	0.5 g L^{-1}	1.5 g L^{-1}	100 mg L^{-1} (100 mL)	100 (30min)	0.060	[4]
Sea urchin-like $NiCo_2O_4$	0.2 g L^{-1}	2.0 g L^{-1}	50 mg L^{-1} (250 mL)	100 (60min)	0.091	This work

Table S2. Comparison of Ea for phenol degradation by different catalyst/PMS systems.

Type of catalyst	Ea (kJ mol^{-1})	Reference
Co/SBA-15-Cl	81.40	[5]
$CoMoO_4$	69.80	[6]
sea urchin-like $NiCo_2O_4$	68.89	This work

Analytical methods for phenol by GC-MS：

The sample pretreatment and test methods of GC-MS method are as follows: Collect 30 mL of sample at a specific reaction time, and then add 30 mL of ethyl acetate to extract the sample. Subsequently, the sample was separated by the separation funnel, and added anhydrous sodium sulfate for dehydration, and finally used a rotary evaporator to concentrate and filter in a 60 °C water bath to obtain the sample to be tested.

The column temperature of GC-MS was set as follows: initially 40 ° C for 2 min, then ramp to 120 °C at 30 ° C min^{-1} rate held for 10 min, and finally ramp to 220 ° C at 40 °C min^{-1} rate held for 10 min (the total analysis time is 31.67 min). The gas chromatographic column is Rtx-5MS (30.0m × 0.25 mm TD, film thickness is 0.25 μm). The whole detection system uses high-purity helium as carrier gas.

References

1. Liao, Z., et al., Degradation of Phenol Using Peroxymonosulfate Activated by a High Efficiency and Stable CoMgAl-LDH Catalyst. Materials, 2019. 12(6).

2. Anbia, M. and M. Rezaie, Synthesis of Supported Ruthenium Catalyst for Phenol Degradation in the Presence of Peroxymonosulfate. Water, Air, & Soil Pollution, 2016. 227(9).

3. Othman, I., et al., Catalytic activation of peroxymonosulfate using $CeVO_4$ for phenol degradation: An insight into the reaction pathway. Applied Catalysis B: Environmental, 2020. 266.

4. Liu, X., et al., Construction of Z-scheme $CuFe_2O_4/MnO_2$ photocatalyst and activating peroxymonosulfate for phenol degradation: Synergistic effect, degradation pathways, and mechanism. Environmental Research, 2021. 200.

5. Shukla, P., et al., Co-SBA-15 for heterogeneous oxidation of phenol with sulfate radical for wastewater treatment. Catalysis Today, 2011. 175(1): p. 380-385.

6. Fan, Y., et al., $CoMoO_4$ as a novel heterogeneous catalyst of peroxymonosulfate activation for the degradation of organic dyes. RSC Advances, 2017. 7(57): p. 36193-36200.
